# Supplementary material for: Defining and estimating effects in cluster randomized trials: A methods comparison
Source: Stat Med. Author manuscript; Available in PMC 2024 Feb 27. (PMC10898620; doi:10.1002/sim.9813)
Supplement: supporting info [file NIHMS1963898-supplement-supporting_info.pdf]

SUPPLEMENTARY MATERIALS: Defining and Estimating Effects in Cluster Randomized Trials: A Methods Comparison

Alejandra Benitez, Maya L. Petersen, Mark J. van der Laan,
Nicole Santos, Elizabeth Butrick, Dilys Walker,
Rakesh Ghosh, Phelgona Otieno, Peter Waiswa, Laura B. Balzer

April 23, 2023

Appendix A: General Definition of Causal Effects in CRTs

Recall $i = \{1, \ldots, N_j\}$ indexes individuals in cluster $j = \{1, \ldots, J\}$. Consider the cluster-level counterfactual outcome defined as the weighted sum of the individual-level counterfactual outcomes: $Y_j^c(a) = \sum_i \alpha_j Y_{ij}(a)$ for some cluster-specific weight α_j. We can further generalize our definition of treatment-specific mean as

$$\Phi^{\star,J}(a) = \frac{1}{J}\sum_{j=1}^{J} \gamma_j Y_j^c(a) \tag{S.1}$$

for user-specified weights such that $\sum_j \gamma_j = J$. For example, setting $\gamma_j = 1$ recovers the cluster-level parameter $\Phi^{c,J}(a)$, as in Eq. 3 in the Main Manuscript. Alternatively, setting $\gamma_j = J/N_T \times 1/\alpha_j$ recovers the individual-level parameter $\Phi^J(a)$, as in Eq. 5 in the Main Manuscript. To illustrate, we again focus on the setting where $\alpha_j = 1/N_j$ is the inverse cluster size. Then we have

$$\frac{1}{J}\sum_{j=1}^{J} \frac{J}{N_T}\frac{1}{\alpha_j} Y_j^c(a) = \frac{1}{J}\sum_{j=1}^{J}\frac{J}{N_T} N_j \sum_{i=1}^{N_j}\frac{1}{N_j} Y_{ij}(a) = \frac{1}{N_T}\sum_{j=1}^{J}\sum_{i=1}^{N_j} Y_{ij}(a) \tag{S.2}$$

Altogether Eq. S.1 allows us to consider a wide range of effects defined at the cluster-level or individual-level, regardless of whether the data are collected at the cluster-level or individual-level.

Appendix B: Hybrid TMLE

Recall that the first step of the cluster-level TMLE (Section 3.1) is to obtain an initial estimator of the conditional expectation of the cluster-level outcome $\mu^c(A, E, W^c)$. Instead of only considering cluster-level approaches, we can expand our candidate estimators by including aggregates of individual approaches [1]. Consider, for example, the following specification of the expected individual-level outcome $\mathbb{E}(Y|A, E, W)$:

$$\mu(A, E, W) = logit^{-1}[\beta_0 + \beta_A A + \beta_E E + \beta_W W]$$

We could estimate these coefficients by pooling participant data across clusters and running an individual-level logistic regression. Afterwards, for each cluster j, we would obtain and summarize the individual-level predicted outcomes to generate a candidate estimator of the expected cluster-level outcome:

$$\hat{\mu}^c(A_j, E_j, W_j^c) = \sum_{i=1}^{N_j} \alpha_{ij} \times \hat{\mu}(A_j, E_j, W_{ij})$$

with the selected α_{ij} corresponding to the relevant cluster-level summary of the individual-level outcomes (i.e., Eqs. 2 and 7 in the Main Manuscript); throughout, we have been focusing on $\alpha_{ij} = 1/N_j$ for $i = \{1, \ldots, N_j\}$. Then estimation and inference would proceed as described in Section 3.1 for the cluster-level TMLE. Thus, this approach naturally targets the cluster-level treatment-specific mean $\Psi_0^c(a) = \mathbb{E}_0[\mathbb{E}_0(Y^c|a, E, W^c)]$, and therefore cluster-level effects, as in Eq. 10 in the Main Manuscript. However, as previously discussed, we can modify the weights to, instead, target an individual-level effect. More importantly, we can now use Adaptive Prespecification to choose between candidate estimators of μ^c based on cluster-level approaches or aggregates of individual-level approaches. In the latter, the initial estimator of μ^c is based on individual-level data that is pooled across clusters; therefore, we could consider methods that are more data-adaptive than GLMs. Specifically, we could use Super Learner, an ensemble machine learning algorithm [2]. Additionally, since the cluster-level TMLE (Section 3.1.2), the Hierarchical TMLE (Section 3.2.1), and the Hybrid TMLE (described here) can target the same effects, we can use Adaptive Prespecification to select the TMLE resulting in the greatest precision for the effect of interest.

Appendix C: Additional Results for Simulation I

Recall in Simulation I, the cluster sizes varied, but the cluster-level relative effect was equivalent to the individual-level relative effect: 0.83. In other words, there was no informative cluster size. While the true values of target effects were identical, their interpretation (Sec. 2.2) and estimator implementation depends on the target. Therefore, we expanded Simulation I to study the finite sample performance of the unadjusted estimator, the cluster-level TMLE, and Hierarchical TMLE when the target of inference was the cluster-level and individual-level relative effect. Additionally, we examined performance with fewer clusters ($J = 10$), smaller clusters ($N_j \sim Norm(20, 10)$ subject to a minimum of 10 participants/cluster), and under the null, where we generated the outcome when setting the intervention terms to zero: $Y_{ij} \sim \mathbb{1}\big[U_{Y_{ij}} < logit^{-1}(-0.75 + 0 \times A_j + 0.8W1_{ij} + 0.4W2_{ij} - 0.3E1_j + 0 \times A_jW2_{ij})\big]$.

Across all settings and as expected, the attained power of the TMLEs was substantially higher than the unadjusted effect estimator (Table S.1 of the Supplementary Materials). Also as expected, a smaller number of randomized units J and smaller cluster sizes (i.e., average N_j) resulted in reduced power. Throughout, the TMLEs maintained good-to-conservative confidence interval coverage. Under the null, the TMLEs also had good-to-conservative Type-I error control in all settings (Table S.2 of the Supplementary Materials). Altogether these additional simulation results illustrate how TMLE can flexibly estimate a variety of causal effects and adaptively adjust for substantial precision gains, while preserving confidence interval coverage and Type-I error control.

Appendix D: Additional Results from Simulation II

In Simulation II, we examined performance under the null, where we generated the outcome by setting the intervention terms to zero: $Y_{ij} \sim \mathbb{1}[U_{Y_{ij}} < logit^{-1}(0.5 + W1_{ij}/6 + W2_{ij}/2 + W3_{ij}/4 + E1_j/5 + E2_j/5 - \tilde{N}_j/8 - 0 \times A\tilde{N}_j/5)]$. The results are given in Table S.3 of the Supplementary Materials and demonstrate the TMLEs with and without Adaptive Prespecification tightly preserved Type-I error control for both the cluster-level and individual-level effect.

Appendix E: Computing code

The simulation study and real data analysis were conducted in `R` (v4.2.1) and utilized the `nbpMatching`, `geesmv`, `CRTgeeDR`, and `ltmle` packages, among others [3–7]. Computing code to reproduce the simulation studies and to analyze the PTBi Study is available at `https://github.com/LauraBalzer/Comparing_CRT_Methods`. As previously noted, an `R` package to estimate effects with TMLE in both individually randomized and cluster randomized trials is under construction: `https://github.com/LauraBalzer/tmle4rcts`.

Table S.1: **Performance of TMLEs for the cluster-level and individual-level relative effects across 500 iterations of Simulation I when there is an effect.**

	Cluster-level effect: $\Psi_0^c(1)/\Psi_0^c(0) = 0.83$						Individual-level effect: $\Psi_0(1)/\Psi_0(0) = 0.83$					
	pt	bias	σ	$\bar{\hat{\sigma}}$	covg	power	pt	bias	σ	$\bar{\hat{\sigma}}$	covg	power
	$J = 20$ clusters of average size N_j=150											
Unadj	0.84	0.01	0.17	0.17	0.97	0.18	0.85	0.02	0.20	0.18	0.95	0.17
C-TMLE	0.83	0.00	0.04	0.05	0.98	0.99	0.83	-0.00	0.04	0.05	0.97	0.98
H-TMLE	0.83	0.00	0.04	0.05	0.98	0.99	0.83	-0.00	0.04	0.05	0.97	0.97
	$J = 10$ clusters of average size N_j=150											
Unadj	0.85	0.03	0.26	0.24	0.96	0.10	0.87	0.04	0.29	0.24	0.93	0.12
C-TMLE	0.83	-0.00	0.06	0.06	0.97	0.79	0.83	-0.00	0.09	0.06	0.95	0.78
H-TMLE	0.83	-0.00	0.06	0.07	0.97	0.75	0.83	-0.00	0.07	0.07	0.95	0.74
	$J = 20$ clusters of average size N_j=20											
Unadj	0.85	0.01	0.19	0.19	0.95	0.14	0.85	0.01	0.19	0.19	0.95	0.13
C-TMLE	0.83	-0.00	0.07	0.07	0.96	0.69	0.83	-0.00	0.07	0.07	0.96	0.73
H-TMLE	0.83	-0.00	0.07	0.07	0.97	0.68	0.83	-0.00	0.07	0.07	0.96	0.71
	$J = 10$ clusters of average size N_j=20											
Unadj	0.86	0.03	0.27	0.26	0.96	0.08	0.87	0.04	0.28	0.26	0.96	0.09
C-TMLE	0.83	-0.01	0.11	0.10	0.94	0.39	0.83	-0.00	0.10	0.09	0.94	0.44
H-TMLE	0.83	-0.01	0.11	0.10	0.95	0.39	0.83	-0.00	0.10	0.09	0.94	0.41

"Unadj" refers to the unadjusted estimator, implemented as a cluster-level TMLE for the effect of interest and without covariate adjustment. "C-TMLE" and "H-TMLE" refer to the cluster-level TMLE and to Hierarchical TMLE, respectively; both were implemented with Adaptive Prespecification to select from $\{\emptyset, W1^c, W2^c\}$ and $\{\emptyset, W1, W2\}$, respectively.
"pt" is the average point estimate; "bias" is the average difference between the point estimate and the target effect; σ is the standard deviation of the point estimates on the log-scale; $\bar{\hat{\sigma}}$ is the average standard error estimate on the log-scale; "covg" is the proportion of times the 95% confidence interval contained the true effect, "power" is the proportion of times the false null hypothesis was rejected.

Table S.2: **Performance of TMLEs for the cluster-level and individual-level relative effects across 500 iterations of Simulation I under the null**

	Cluster-level effect: $\Psi_0^c(1)/\Psi_0^c(0)$ =1.0						Individual-level effect: $\Psi_0(1)/\Psi_0(0) = 1.0$					
**Null**	pt	bias	σ	$\bar{\hat{\sigma}}$	covg	Type-I	pt	bias	σ	$\bar{\hat{\sigma}}$	covg	Type-I
$J = 20$ clusters of average size N_j=150												
Unadj	1.01	0.01	0.16	0.16	0.96	0.04	1.02	0.02	0.18	0.17	0.95	0.05
C-TMLE	1.00	0.00	0.03	0.04	0.99	0.01	1.00	-0.00	0.03	0.03	0.98	0.02
H-TMLE	1.00	0.00	0.03	0.04	0.98	0.02	1.00	-0.00	0.03	0.04	0.98	0.02
$J = 10$ clusters of average size N_j=150												
Unadj	1.03	0.03	0.24	0.22	0.97	0.03	1.05	0.05	0.27	0.23	0.93	0.07
C-TMLE	1.00	0.00	0.05	0.05	0.98	0.02	1.00	0.00	0.05	0.04	0.97	0.03
H-TMLE	1.00	0.00	0.05	0.05	0.98	0.02	1.00	0.00	0.05	0.05	0.97	0.03
$J = 20$ clusters of average size N_j=20												
Unadj	1.01	0.01	0.17	0.17	0.94	0.06	1.02	0.02	0.18	0.18	0.95	0.05
C-TMLE	1.00	-0.00	0.06	0.06	0.97	0.03	1.00	-0.00	0.06	0.05	0.96	0.04
H-TMLE	1.00	-0.00	0.06	0.06	0.97	0.03	1.00	-0.00	0.06	0.06	0.96	0.04
$J = 10$ clusters of average size N_j=20												
Unadj	1.04	0.04	0.25	0.24	0.97	0.03	1.05	0.05	0.26	0.24	0.97	0.03
C-TMLE	1.00	0.00	0.09	0.08	0.94	0.06	1.00	0.00	0.08	0.07	0.94	0.06
H-TMLE	1.00	0.00	0.09	0.08	0.95	0.05	1.00	0.00	0.08	0.07	0.94	0.06

"Unadj" refers to the unadjusted estimator, implemented as a cluster-level TMLE for the effect of interest and without covariate adjustment. "C-TMLE" and "H-TMLE" refer to the cluster-level TMLE and to Hierarchical TMLE, respectively; both were implemented with Adaptive Prespecification to select from $\{\emptyset, W1^c, W2^c\}$ and $\{\emptyset, W1, W2\}$, respectively.
"pt" is the average point estimate; "bias" is the average difference between the point estimate and the target effect; σ is the standard deviation of the point estimates on the log-scale; $\bar{\hat{\sigma}}$ is the average standard error estimate on the log-scale; "covg" is the proportion of times the 95% confidence interval contained the true effect, and "Type-I" is the proportion of times the true null hypothesis was rejected.

Table S.3: **Performance of TMLEs for the cluster-level and individual-level relative effects across 500 iterations in Simulation II under the null**

	Cluster-level effect: $\Psi_0^c(1)/\Psi_0^c(0) = 1$						Individual-level effect: $\Psi_0(1)/\Psi_0(0) = 1$					
	pt	bias	σ	$\bar{\hat{\sigma}}$	covg	Type-I	pt	bias	σ	$\bar{\hat{\sigma}}$	covg	Type-I
C-TMLE	1.01	0.01	0.09	0.10	0.98	0.02	1.01	0.01	0.11	0.12	0.96	0.04
C-TMLE-AP	1.01	0.01	0.07	0.09	0.98	0.02	1.01	0.01	0.09	0.10	0.96	0.04
H-TMLE	1.01	0.01	0.09	0.11	0.98	0.02	1.01	0.01	0.10	0.12	0.97	0.03
H-TMLE-AP	1.01	0.01	0.07	0.09	0.99	0.01	1.01	0.01	0.08	0.10	0.98	0.02

"C-TMLE" and "H-TMLE" refer to the cluster-level TMLE and to Hierarchical TMLE, respectively. Both were implemented with fixed or adaptive adjustment via Adaptive Prespecification ("-AP").
"pt" is the average point estimate; "bias" is the average difference between the point estimate and the target effect; σ is the standard deviation of the point estimates on the log-scale; $\bar{\hat{\sigma}}$ is the average standard error estimate on the log-scale; "covg" is the proportion of times the 95% confidence interval contained the true effect, and "Type-I" is the proportion of times the true null hypothesis was rejected.

References

[1] Laura Balzer et al. “A new approach to hierarchical data analysis: Targeted maximum likelihood estimation for the causal effect of a cluster-level exposure”. In: *Statistical Methods in Medical Research* 28.6 (2019), pp. 1761–1780.

[2] Mark van der Laan, Eric C. Polley, and Alan E. Hubbard. “Super Learner”. In: *Statistical Applications in Genetics and Molecular Biology* 6.1 (2007).

[3] R Core Team. *R: A Language and Environment for Statistical Computing*. R Foundation for Statistical Computing. Vienna, Austria, 2020. URL: `http://www.R-project.org`.

[4] Cole Beck, Bo Lu, and Robert Greevy. *nbpMatching: Functions for Optimal Non-Bipartite Matching*. R package version 1.5.1. 2016. URL: `https://CRAN.R-project.org/package=nbpMatching`.

[5] Ming Wang. *geesmv: Modified Variance Estimators for Generalized Estimating Equations*. R package version 1.3. 2015. URL: `https://CRAN.R-project.org/package=geesmv`.

[6] M. Prague. *CRTgeeDR: Doubly Robust Inverse Probability Weighted Augmented GEE Estimator*. R package version 2.0. 2017. URL: `https://CRAN.R-project.org/package=CRTgeeDR`.

[7] S.D. Lendle et al. “ltmle: An R Package Implementing Targeted Minimum Loss-based Estimation for Longitudinal Data”. In: *Journal of Statistical Software* 81.1 (2017), pp. 1–21.
